# Neuromuscular and proprioceptive exercises for knee osteoarthritis: building balance, stability, and patient confidence

**DOI:** 10.1093/jscr/rjaf1011

**Published:** 2025-12-23

**Authors:** Sarah S Joseph, George Ampat

**Affiliations:** Free From Pain Clinic, 681, Liverpool Road, Ainsdale, Southport PR8 3NS, United Kingdom; Trauma and Orthopaedics, School of Medicine, University of Liverpool, Liverpool, L69 3GE, United Kingdom

**Keywords:** knee, osteoarthritis, proprioception, neuromuscular exercise

## Abstract

Knee osteoarthritis (OA) is a degenerative joint disease characterized by pain, reduced mobility, and diminished quality of life. While surgical interventions and joint injections are common, exercise remains central to non-operative management. However, increased pain during exercise can lead to non-compliance, often due to instability. Therefore, any exercise regimen should address this instability to improve compliance. The ARISE neuromuscular program is a proprioceptive-based exercise program designed to address instability while increasing strength. A 67-year-old male with knee OA, initially seeking an injection, was counseled to use the ARISE program. At the 3-month follow-up, his pain reduced from 7/10 to 2/10, and his sit-to-stand performance improved from 10 to 17 repetitions within 30 s. This case supports the use of non-operative, proprioceptive-based neuromuscular programs to improve function, reduce symptoms, and potentially reduce the need for surgical intervention in individuals with knee OA.

## Introduction

Knee osteoarthritis (OA) is a leading cause of disability worldwide, affecting over 10 million people in the UK [1]. It is characterized by joint pain, stiffness, and reduced mobility due to cartilage degeneration, subchondral changes, and synovial inflammation [2]. While surgical treatments and joint injections are common, exercise is key in the non-surgical management of knee OA. However, pain during exercise, often due to joint instability, can reduce participation in exercise programs. Therefore, exercise programs should address this instability to improve engagement and enhance outcomes during non-operative management.

Non-surgical strategies, particularly strengthening or resistance exercises, are recommended for symptom relief and functional improvement in knee OA [3, 4]. However, some non-operative programs emphasize proprioceptive training to improve joint position sense and control, components often underrepresented in standard approaches despite their importance in restoring joint function [5]. Neuromuscular training targets these deficits by enhancing joint stability and improving movement coordination, ultimately leading to better outcomes in pain reduction and daily function [6, 7]. The ARISE framework—Awareness, Resistance, Individualized, Stability, Essential—offers a structured, personalized approach, combining proprioceptive retraining, resistance exercises, and functional movement strategies. This case report describes the outcomes of a patient with symptomatic knee OA managed through the ARISE program, highlighting the benefits of neuromuscular rehabilitation in reducing pain and improving function.

## Case report

A 67-year-old retired man presented with a 6-month history of gradually worsening left knee pain and reduced walking endurance. Although previously active, he now experienced fatigue and needed to rest after walking for ~20 min. He had received a hyaluronic acid injection in the past, which provided only short-term relief. Examination revealed that he stood with an erect posture and walked independently, though occasional facial expressions indicated discomfort. The range of motion in his knee was limited to 0–90° due to pain. On palpation, there was tenderness on the medial and lateral joint lines of his left knee. No crepitus was present, and the femoral stretch test was positive. Neurological examination was normal.

**Figure 1:**
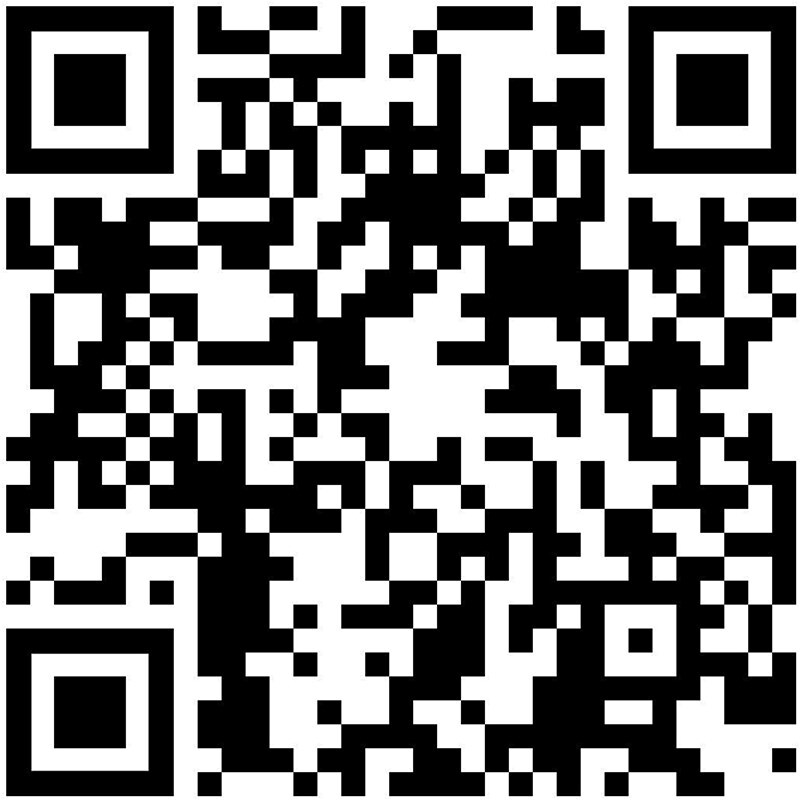
QR code providing the YouTube video by the senior author detailing the neuromuscular exercises for knee pain.

**Figure 2:**
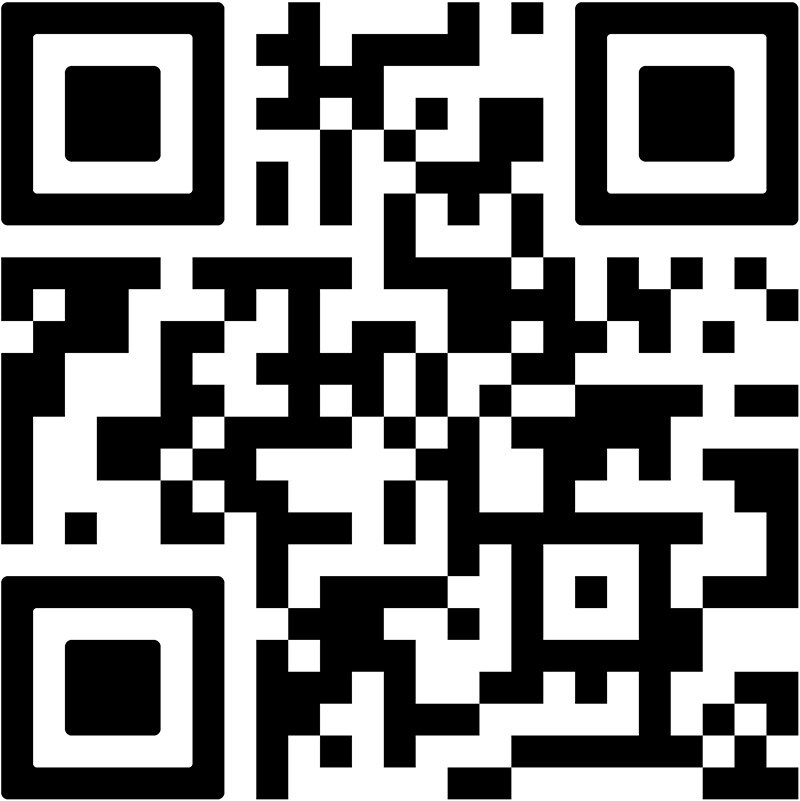
QR code linking to a YouTube video of the patient testimonial.

He was prescribed a neuromuscular exercise program adapted by the senior author (https://youtu.be/HN_JQYpzpHU) (Fig. 1). This program emphasized proprioception, muscle strengthening, and core stability to improve pain and function. Co-codamol was recommended for symptom control. At the 3-month follow-up, pain decreased from 7/10 to 2–3/10, and 30-s sit-to-stand repetitions improved from 12 to 17. He has remained consistent with the program and continues to experience ongoing benefits (https://youtu.be/P-Ms9tFLa6A) (Fig. 2).

## Discussion

Knee OA is increasingly recognized as a condition influenced by more than joint degeneration alone. Deficits in neuromuscular control and proprioception are key contributors to pain, instability, and functional limitation [2]. Proprioceptive decline in OA affects joint position sense and motor control, resulting in altered loading patterns and increased symptom sensitivity [3]. Despite this, these issues are rarely addressed in standard strength-based rehabilitation programs.

The ARISE program addresses this gap by integrating proprioceptive retraining as the foundation of its intervention. Its structure is guided by three physiological elements essential to proprioception: Pressure (graded load), Position (alignment and joint sense), and Pace (controlled timing). By prioritizing early joint awareness and neuromuscular control before progressing to strengthening, ARISE aims to minimize symptom aggravation and facilitate safer, more gradual rehabilitation.

The GLA:D® program is a similar, well-established, evidence-based non-surgical intervention for knee OA, combining structured education with supervised neuromuscular exercise to improve strength, joint control, and self-management [8]. Exercise-based approaches, including aerobic exercise, resistance training, and aquatic therapy, have all been shown to reduce pain and improve physical function in individuals with knee OA. A review by Mo concluded that both aerobic and resistance programs are effective in improving mobility and alleviating symptoms [9]. Aerobic exercise, such as walking, cycling, or swimming, enhances cardiovascular health, endurance, and fitness while minimizing joint strain. Aquatic therapy uses water’s buoyancy to reduce joint impact, allowing for movements with less pain and a better range of motion [10]. However, these programs often lack components to address proprioceptive deficits.

Furthermore, neuromuscular programs like ARISE incorporate proprioceptive elements to improve movement quality and joint control. These programs are designed to avoid excessive or abrupt loading by promoting smooth, deliberate movements—particularly important for individuals with OA-related instability or poor coordination. Rather than focusing solely on isolated muscle strengthening, this approach promotes functional movement patterns that are more relevant to everyday activities [11].

Providing further evidence for this approach, Richhariya found that integrating proprioceptive exercises and core strengthening with conventional physiotherapy led to greater reductions in pain and improvements in quality of life compared to usual care alone [12]. By addressing not only strength but also sensorimotor control and postural stability, ARISE presents a more holistic alternative to general protocols—especially for those needing improved balance, movement control, and confidence in daily function.

While long-term outcomes and neurophysiological mechanisms remain to be fully established, current evidence and this case suggest that ARISE is a promising, patient-centered intervention. Its focus on proprioception and neuromuscular control may support better functional outcomes and enhance exercise tolerance in individuals with knee OA.
